# PANCREATICODUODENECTOMY: IMPACT OF THE TECHNIQUE ON OPERATIVE OUTCOMES AND SURGICAL MORTALITY

**DOI:** 10.1590/0102-672020180001e1412

**Published:** 2019-01-07

**Authors:** Achiles Queiroz de Monteiro REZENDE, João Paulo Simões DUTRA, Martinho Antonio GESTIC, Murillo Pimentel UTRINI, Francisco CALLEJAS-NETO, Elinton Adami CHAIM, Everton CAZZO

**Affiliations:** 1 Department of Surgery, Faculty of Medical Sciences, State University of Campinas, Campinas, SP, Brazil

**Keywords:** Pancreaticoduodenectomy, Ampulla of Vater, Pancreas, Pancreatitis, chronic, Pancreatic neoplasms, Pancreaticoduodenectomia, Ampola hepatopancreática, Pâncreas, Pancreatite crônica, Neoplasias pancreáticas

## Abstract

**Background:**

Pancreaticoduodenectomy (PD) is a procedure associated with significant morbidity and mortality. Initially described as gastropancreaticoduodenectomy (GPD), the possibility of preservation of the gastric antrum and pylorus was described in the 1970s.

**Aim:**

To evaluate the mortality and operative variables of PD with or without pyloric preservation and to correlate them with the adopted technique and surgical indication.

**Method:**

Retrospective cohort on data analysis of medical records of individuals who underwent PD from 2012 through 2017. Demographic, anthropometric and operative variables were analyzed and correlated with the adopted technique (GPD vs. PD) and the surgical indication.

**Results:**

Of the 87 individuals evaluated, 38 (43.7%) underwent GPD and 49 (53.3%) were submitted to PD. The frequency of GPD (62.5%) was significantly higher among patients with pancreatic neoplasia (p=0.04). The hospital stay was significantly shorter among the individuals submitted to resection due to neoplasias of less aggressive behavior (p=0.04). Surgical mortality was 10.3%, with no difference between GPD and PD. Mortality was significantly higher among individuals undergoing resection for chronic pancreatitis (p=0.001).

**Conclusion:**

There were no differences in mortality, surgical time, bleeding or hospitalization time between GPD and PD. Pancreas head neoplasm was associated with a higher indication of GPD. Resection of less aggressive neoplasms was associated with lower morbidity and mortality.

## RESUMO

**Racional:**

A duodenopancreatectomia (DP) é procedimento associado com significativa morbimortalidade. Inicialmente descrita como gastroduodenopancreatectomia (GDP), a possibilidade de preservação do antro gástrico e piloro foi descrita na década de 1970.

**Objetivo:**

Avaliar a mortalidade e variáveis operatórias da DP com ou sem preservação pilórica e correlacioná-las com a técnica adotada e indicação cirúrgica.

**Método:**

Estudo de coorte histórica, baseado em análise de dados de registros médicos de indivíduos submetidos à DP entre os anos de 2012 a 2017. Foram analisadas variáveis demográficas, antropométricas e operatórias e correlacionadas com a técnica adotada (GDP *vs.* DP) e a indicação cirúrgica.

**Resultados:**

Dos 87 indivíduos avaliados, 38 (43,7%) foram submetidos à GDP e 49 (53,3%) à DP. A frequência de realização da GDP (62,5%) foi significativamente maior entre os pacientes com neoplasia de pâncreas (p=0,04). O tempo de internação total foi significativamente menor entre os indivíduos submetidos à ressecção por neoplasias de comportamento menos agressivo (p=0,04). A mortalidade cirúrgica foi de 10,3%, não havendo diferença entre GDP e DP. A mortalidade foi significativamente maior entre os indivíduos submetidos à ressecção por pancreatite crônica (p=0,001).

**Conclusão:**

Não houve diferenças na morbimortalidade, tempo cirúrgico, sangramento ou tempo de internação entre GDP e DP. A neoplasia de cabeça de pâncreas associou-se mais com indicação de GDP. A ressecção de neoplasias menos agressivas associou-se a menor morbimortalidade.

## INTRODUCTION

Pancreaticoduodenectomy (PD) is defined as the resection of the pancreas head and the entire duodenum, associated with anatomical structures closely located and/or whose blood flow is affected by the procedure. Usually, the intrapancreatic and supraduodenal portions of the common bile duct and the gallbladder are also resected; the gastric antrum may be resected or not, depending on the technique adopted.

**FIGURE 1:**
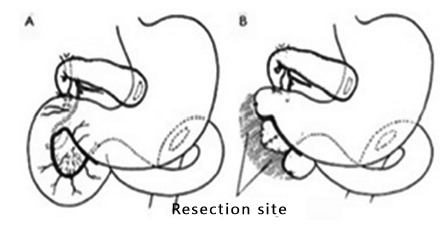
Two-step pancreaticoduodenectomy: A) cholecystogastrostomy and gastrojejunostomy; B) ressection of the head of pancreas, duodenum and main bile duct (Adapted from Whipple et al.^27^)

The first well-succeeded partial PD was described in 1909 by a German surgeon named Kausch. He proposed a 2-step operation. Firstly, a cholecystojejunostomy and a Brown enteric anastomosis were performed; their objective was to drain and decompress the biliary tree and, after two months, the em-bloc resection of the distal stomach, proximal duodenum and head of pancreas was carried out, followed by the reconstruction with a loop gastrojejunostomy and na end-to-end pancreatojejunostomy^10^ ^,^ ^15^. Only in 1935, Whipple successfully reproduced the previously described procedure, also in a 2-step approach. It began with a cholecystogastrostomy, which evolved to a Roux-em-Y cholecystojejunostomy. The resection was carried out later, however with the closure of the main pancreatic duct (Figure 1)^10^ ^,^ ^26^.

**FIGURE 2:**
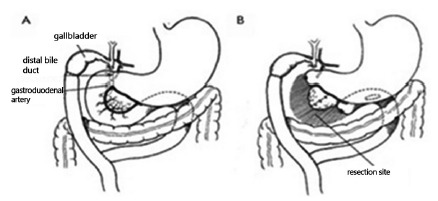
Two-step revision of the surgery proposed by Whipple in 1938: A) Roux-en-Y cholecystojejunostomy; B) resection of the pancreas head, duodenum and common bile duct (Adapted from Whipple^28^)

**FIGURE 3:**
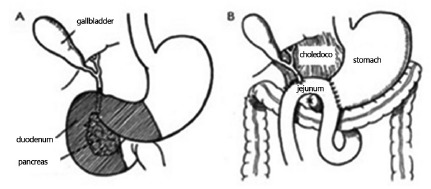
Single-step operation: A) resection site; B) single-loop reconstruction with three anastomoses (Adapted from Whipple^29^)

In 1940, Whipple performed the first single-step PD in a patient with a presumptive diagnosis of gastric cancer, whose intraoperative findings disclosed a neoplasm in the head of pancreas. Since this individual did not present jaundice, it was opted to perform the surgery through a single procedure (Figure 2)^10^ ^,^ ^27^ ^,^ ^28^. In 1942, Whipple finally adopted an end-to-end duct-mucous pancreaticojejunostomy and described his classic procedure, which was named gastropancreatoduodenectomy (GPD, Figure 3)^10^ ^,^ ^29^.

In 1978, Traverso and Longmire published two cases of pylorus-sparing PD, according to the previous description of Watson in 1944; one case for a benign disease and the other due to cancer in the third portion of the duodenum^10^ ^,^ ^14^. It was then suggested that the preservation of the gastric antrum and pylorus could not only decrease the operative time, but also could lead to better postoperative outcomes, since there was a maintenance of the gastrointestinal tract function, with the possibility of a better long-term nutritional status, and lower incidences of jejunal ulcers and dumping syndrome^14^.

During the 1960 and 70 decades, the mortality among individuals who underwent PD was approximately 25%. By 1980-90, due to advances of the surgical technique and resources, associated with the rapid development of intensive postoperative care, allied to the advent and organization of excellence, the mortality rate following PD with or without preservation of the pylorus gradually decreased to levels under 10%^10^ ^,^ ^14^. It is important to take into consideration that the only potentially curative treatment for periampullary and head of pancreas tumors is the surgical resection and that, even when associated with adjuvant chemotherapy, the 5-year survival rate for pancreatic cancer reaches about 21%. Better late results are observed among individuals with neoplasms that present a less aggressive behavior, such as neuroendocrine and ampullary tumors^14^ ^,^ ^16^. Hence, a relevant concern in regards to the treatment of the periampullary tumors refers to the surgery and its complications, since both morbidity and mortality, despite recent advances, remain significant. Several studies point out that between 30-50% of the individuals who undergo a pancreatic resection do not present conditions sufficient to undergo na adjuvant therapy, and one of the major reasons for this contraindication is the postoperative clinical deterioration or the delay to be referred caused by operative complications^1^ ^,^ ^30^.

A number of studies comparing the classic operation (GPD) with the pylorus-sparing variation (PD) did not demonstrate significant differences in regards to survival time and postoperative mortality, only lower operative time and intraoperative blood loss^13^ ^,^ ^14^ ^,^ ^16^.

Nonetheless, Roder et al. ^20^, in a prospective non-randomized study, observed better survival rates among individuals who underwent GPD due to ductal adenocarcinoma of the head of pancreas. In the same study, this advantage was not observed among individuals with periampullary neoplasms. In 2013, Leichtle et al. also compared the outcomes of GPD and PD, through an analysis of the ACS NSQIP database, with a total of 6988 operations with or without preservation of the pylorus. There were no significant differences in regard to morbimortality; they only observed lower sugical time, need for blood transfusion and hospital stay in the PD group^16^.

Recently, Hüttner et al. performed a meta-analysis which enrolled individuals who underwent PD or GPD; no differences were observed in regard to morbimortality, although the high heterogeneity and varied quality of the selected studies were highlighted^13^.

The current study aimed to evaluate the mortality and operative outcomes of PD with or without preservation of the pylorus, and correlate them with the adopted technique and the indication of the procedure.

## METHODS

### Study design

This is a descriptive observational retrospective study classified as a historical cohort, based on the analysis of data from medical records of individuals who underwent PD from 2012 through 2017 in the ^1^Department of Surgery, Faculty of Medical Sciences, State University of Campinas, Campinas, SP, Brazil, due to periampullary diseases. It was performed by means of an electronic research in the Service of Medical Archives. The study protocol underwent an ethical evaluation and was approved by the local institutional Review Board under the reference 2.241.785/CAAE: 72739317.2.0000.5404.

### Inclusion criteria

This protocol included individuals: 1) who underwent surgical resection due to tumors of pancreas, duodenal ampulla, and distal bile duct, cystic lesions, solid pseudopapillary tumors, chronic pancreatitis, and neuroendocrine tumors; 2) with a confirmed histopathological diagnosis; 3) of both genders; 4) aged 18 years or older; 5) which underwent PD with or without preservation of the pylorus.

### Exclusion criteria

Were excluded individuals: 1) from vulnerable groups (underaged, mentally impaired, and institutionalized); 2) who underwent surgery due to recidivism of loco-regional invasion of tumors from other non-periampullary sites; 3) whose medical records were incomplete or absent.

### Variables

The variables and concepts adopted were: 1) age expressed in years; 2) gender expressed in male or female; 3) weight, height, and body mass index; 4) histological type and histopathological variables; 5) operative time expressed in minutes; 6) estimated blood loss expressed in ml; 7) intensive care length of stay expressed in days; 8) length of hospital stay expressed in days; 9) 30-day surgical morbidity; 10) 30-day surgical mortality.

### Surgical technique

All the procedures were performed under the same command and supervision of one of the authors. The technique was carried out as follows: 1) opening of the abdominal wall through a bilateral transvesal subscostal Chevron incision; 2) inventary of the cavity; 3) liberation of the duodenum by means of a Kocher maneuver; 4) cholecystectomy and isolation of hepatic hilum elements; 4) section of the main bile duct and isolation of the suprapancreatic portion of the portal vein; 5) isolation of the infrapancreatic portion of the superior mesenteric vein; 6) section of the duodenum (if pylorus-sparing PD) or gastric antrum (if GPD); 7) Whipple’s maneuver and isolation of the splenoportal junction; 8) section of the proximal jejunum; 9) section of the pancreas in the head-to-body transition and en-bloc resection; 10) extended lymphadenectomy - hepatic hilum, celiac trunk, splenic artery, superior mesenteric artery, infra and supra-pyloric lymph nodes; 11) reconstruction: single-loop (duodenojejunostomy, hepaticojejunostomy and pancreaticojejunostomy) or double-loop (gastrojejunostomy or duodenojejunostomy, hepaticojejunostomy, pancreaticojejunostomy and enteroenterostomy); 12) abdominal wall closure.

### Study population

After the electronic research, 99 patients were identified, of whom 10 were excluded because have undergone other surgical procedures (eight cases of palliative biliodigestive shunts, an operation due to gastric cancer metastasis and a Frey operation due to chronic pancreatitis); two were excluded due to incomplete medical records. Hence, the data from 87 individuals were included in this study.

### Statistical analysis

It was performed a descriptive analysis with frequency tables for categorical variables and measures of position and dispersion for continous variables. For the comparison of proportions, it was used the chi-square test or the Fisher’s exact test, when necessary. For the comparison of ordinal or continous measures, it was used the Mann-Whitney test. The level of significance adopted was 5% (p<0.05). For the performance of the analyses, it was used the software SAS System for Windows (Statistic Analysis System), versão 9.2, SAS Institute Inc., 2002-2008, Cary, NC, USA.

## RESULTS

Of 87 individuals evaluated, 38 (46.7%) underwent GPD and 49 (53.3%) PD. There was a predominance of females (60.9%), and the mean age was 59.3 years e the mean BMI was 24.8 kg/m^2^. The individuals who underwent GPD presented a significantly higher BMI than those who underwent PD (p=0.005). There were no differences in regards to age and gender. The demographic and anthropometric data are presented in Table 1.

**TABLE 1:** Comparison of demographic, anthropometric, and surgical variables and mortality between GPD and PD

	GPD	PD	p
Age (years)	61.6 ± 8.4	57.5 ± 13.4	0.1
Gender	Female: 25 (65.8%) Male: 13 (34.2%)	Female: 28 (57.1%) Male: 21 (42.9%)	0.2
BMI (kg/m^2^)	26.4 ± 5	23.6 ± 3.6	0.005
Operative time (minutes)	311 ± 40.6	297.8 ± 56.5	0.3
Estimated blood loss (mL)	957.5 ± 676.8	772 ± 565.8	0.2
Length of stay in intensive care (days)	9.6 ± 12.2	6.7 ± 4.6	0.2
Length of hospital stay (days)	16.2 ± 13.1	10.1 ± 5.7	0.4
Reoperations - n (%)	6 (15.8%)	5 (10.2%)	0.4
Perioperative mortality - n (%)	4 (10.5%)	5 (10.2%)	0.9

**FIGURE 4:**
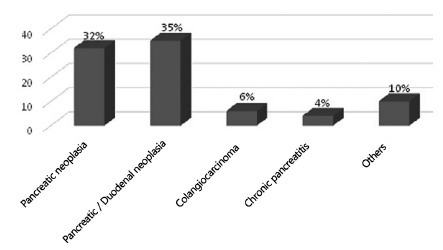
Surgical indications of the pancreaticoduodenectomies

The main surgical indications were head of pancreas cancer (36.7%), duodenum and duodenal ampulla cancer (40.2%), distal cholangiocarcinoma (6.8%), chronic pancreatitis (4.5%) and other neoplasms (intraductal papillary mucinous neoplasm, solid pseudopapillary tumor and neuroendocrine tumors) (11.4%, Figure 4).

Analyzing the procedures performed according to the surgical indication, it was observed that among the individuals who underwent resection due to pancreas cancer, the frequency of GPD (62.5%) was higher than among the other indications (p=0.04). Among the individuals who underwent resection for less aggressive tumors, there was a significant predominance of younger individuals, whose mean age was 44 years old (p<0.001). Table 2 presents the demographic variables compared according to the indication of the procedure.

**TABLE 2:** Comparison of demographic, anthropometric, and surgical variables among the operated individuals according to the etiology

	Pancreas adenocarcinoma	Ampulla/Duodenum cancer	Cholangiocarcinoma	Less aggressive tumors	Chronic pancreatitis	p
n	32	35	6	10	4	N/A
Operation	GDP: 20 DP: 12	GDP: 14 DP: 21	GDP: 3 DP: 3	GDP: 1 DP: 9	GDP: 1 DP: 3	0.040839
Age (years)	59.3 ± 9.1	62.4 ± 9.8	61.7 ± 10	44.1 ± 15.9	67.3 ± 9.5	<0.0001
Gender	M: 10 (31.3%) F: 22 (68.7%)	M: 15 (42.9%) F: 20 (57.1%)	M: 3 (50%) F: 3 (50%)	M: 4 (40%) F: 6 (60%)	M: 2 (50%) F: 2 (50%)	NS
BMI (kg/m^2^)	25.9 ± 5.1	24.5 ± 4.2	23.4 ± 3.1	24.2 ± 3.4	22.2 ± 1.7	NS
Operative time (minutes)	313.4 ± 60.6	307.9 ± 44.9	299.2 ± 59.1	294.5 ± 3.8	293.8 ± 47.8	NS
Blood loss (ml)	1056.9 ± 751.1	787.1 ± 575.1	616.7 ± 116.9	810 ± 440.8	612.5 ± 283.9	NS
Intensive care unit length of stay (days)	7.1 ± 8	9.2 ± 10.8	6.2 ± 4.2	5.7 ± 3.1	12 ± 11.4	NS
Length of hospital stay (days)	10.2 ± 9.7	15.7 ± 10.4	10.1 ± 9.8	7.3 ± 6.4	15.4 ± 13.9	0.0412
Reoperations (n(%))	3 (9.4%)	6 (17.1%)	0	0	2 (50%)	NS
Surgical mortality (n (%))	2 (6.2%)	4 (11.4%)	1 (16.7%)	0	2 (50%)	0.011209

There was no significant differences in regards to surgical time or blood loss in both the comparisons according to the performed procedure (GPD vs. PD) or to the surgical indication.

The overall frequency of reoperations was 12.6%, and there was no difference in this regard between GPD and PD, as well as in relation to the length of stay in the intensive care unit or length of the hospital stay. Comparing the etiologies, the overall hospital stay was lower among those who underwent surgery due to less aggressive tumors (p=0.04). The 30-day surgical mortality was 10.3 %, and there was no significant difference between GPD and PD. Comparing the surgical mortality according to the surgical indication, there was a significantly higher mortality among the individuals who underwent resection due to chronic pancreatitis (p=0.001); among those who underwent surgery due to less aggressive tumors, there was no mortality. Table 1 presents the comparison of the morbimortality variables between GPD and PD groups, whereas Table 2 presents the comparison of these variables according to the surgical indication.

## DISCUSSION

This study considered the early morbidity and mortality outcomes among individuals who underwent PD with or with the preservation of the pylorus, comparing and analyzing the main surgical results according to the main surgical indications of the service.

Comparing the outcomes observed after GPD or PD, there were no significant differences in any of the surgical variables analyzed, with a mean estimated blood loss of 817 ml and a mean surgical time of 304 min; there was also no differences in the early morbidity or mortality between the two surgical modalities; the overall 30-day mortality was 10.3%. In the literature, there is no difference in morbidity or mortality comparing GPD and PD; the reported mortality is the majority of the series is close to the observed in this study^2^ ^,^ ^6^ ^,^ ^18^. Farges et al.^5^, evaluating 22,366 individuals who underwent this procedure in a French national database, observed an overall mortality of 8.1%, whereas Swanson et al.^21^, in a study that analyzed a national USA database comprised of 21,482 patients, reported a surgical mortality of 8%. Both authors reported that mortality rates were significantly higher in services of lower volume, reaching four times higher in hospitals whose volume was less than five annual procedures.

These data confirm the findings of the landmark study of Finks et al.^7^, which analyzed the influence of surgical volume over perioperative morbidity and mortality of Medicare users in the USA, observing a 67% reduction of the mortality after pancreatectomy when it was performed at high-volume centers. Hata et al.^12^, in a meta-analysis, demonstrated that the risk of perioperative mortality after PD was 2.4 times higher in hospitals where less than 30 operations per year were carried out. Such a relevant aspect within these comparison is also the overall profile of the analyzed populations, since the international studies cited here were performed in developed countries, whose social-economical level of development tends to be less compromised than among our population. Evaluating Brazilian data, the surgical outcomes present a tendency to higher morbidity and mortality. Rocha et al.^19^, in a 41-patient series, reported a morbidity of 58% and a surgical mortality of 22%. Wanderlay et al.^24^, analyzing 21 patients, observed a morbidity of 21.7% and mortality of 17.3%. Torres et al.^22^, in a 39-patient series, observed a 30-day mortality of 10.2%^21^ ^-^ ^23^.

More recent studies also did not observe differences of morbidity and mortality comparing GPD and PD; some authors observed lower operative time, need for transfusion and hospital stay among individuals who underwent PD^13^ ^,^ ^14^ ^,^ ^16^.

Within our series, 53.3% of the individuals underwent PD and 46.7% à GPD. Comparing the etiologies, the majority of the individuals with pancreas adenocarcinoma underwent GPD. Moreover, the mean BMI of the individuals who underwent GPD was higher than the observed in the PD group. These findings are likely to be related to the necessity of more aggressive oncologic resections in the individuals with pancreatic adenocarcinoma, in order to obtain free margins and an appropriated locoregional lymph node resection, frequently compromising the blood flow to the distal stomach and proximal duodenum and requiring the resection of these structures. Huttner et al.^13^ published in 2016 a meta-analysis in the Cochrane database which enrolled 512 patients and demonstrated that there was no difference in regards to oncologic outcomes for both procedures. Nevertheless, Roder et al.^20^ observed, in a prospective non-randomized study, a better survival among individuals who underwent GPD due to pancreatic cancer. Furthermore, another likely justification for the predominance of this technique in this population in the higher prevalence of diabetes among the individuals with pancreas cancer and/or the higher BMI, since both these factors are known to co-jointly lead to a higher risk of gastric emptying delay in the postoperative period in individuals who undergo PD^4^ ^,^ ^17^ ^,^ ^25^ ^,^ ^31^. El-Nakeeb et al.^4^ published in 2015 a retrospective study enrolling 588 individuals who underwent PD and evaluated the risk factors that predicted the severity of gastric emptying delay. They observed that obesity and diabetes were significant risk factor, emphasizing the diabetes was an independent predictor. Similarly, the incidence of moderate to severe gastric emptying delay (degrees B or C) was also significantly higher in this group. The gastric emptying delay is described as primary or secondary, and the latter is the commonest, defined as the delay caused by any clinical postoperative complication, such as cavitary abscesses and mainly pancreatic leaks^4^ ^,^ ^17^ ^,^ ^25^ ^,^ ^31^.

Venkat et al.^23^ performed a prospective study comparing individuals who underwent pancreatic resections for ductal adenocarcinoma divided into two groups: one under 45 years old (n=75) and the other abobe this age (n=870), analyzing the type of resection, the tumor staging, and the co-morbidities (CACI) between the groups. As a result, it was observed a lower rate of complications (pancreatic leak and gastric empyting delay) and higher survival among the younger individuals. This finding demonstrated a correlation between a more favorable evolution and the preoperative clinical conditions of the individuals with more physiological reserve. The significant finding of null mortality among the individuals who underwent surgery due to less aggressive tumors is likely to have been observed not only due to the etiology itself, but mainly due to the better clinical status of the patients, since they were younger than the ones with the other etiologies. Another important factor is that there is no necessity for a higher surgical radicality within this group, since these tumors usually do not feature such a locally invasive presentation.

The mortality rate among individuals who underwent PD due to pancreatitis was 50%. All the cases were indicated due to pseudotumoral pancreatitis, where it was not possible to exclude pre or intraoperatively the diagnosis of a concomitant neoplasm. A specific limitation of the analyzed service is the unavailability of an ecoendoscopy equipment to permit a more appropprite preoperative assessment. However, since it is a small group of patients, it is not possible to fully analyze this isolated result. In the literature, the pancreatic resection by means of PD or GPD still presents a role, mainly in cases where is not possible to fully exclude an associated cancer; hybrid techniques which partially resect the head of pancreas, such as Beger of Frey operations, are also adequate for such cases. In regards to surgical outcomes in excellence centers, both modalities present low mortality and similar long-term results^3^ ^,^ ^11^. The pain relief stands next to 80% for both procedures; however, there is a tendency to more functional insufficiencies among the individuals who undergo PD. In the service where this study was developed, the individuals with chronic pancreatitis who present surgical indication undergo decompressive or hybrid procedures: Partington-Rochelle for the cases with chronic pancreatitis with ductal dilatation and no significant involvement of the pancreas head, and Frey surgery for those with greater involvement of the pancreas head. The outcomes observed after those procedures are highly satisfactory, leading to high rates of success to long-term pain relief (91.4%), overall morbidity of 28.7% e null mortality, according to Gestic et al^8^ ^,^ ^9^.

## CONCLUSION

There were no significant differences in regards to morbidity or mortality between GPD and PD. The head of pancreas cancer was associated with a higher indication of GPD. The resection of less aggressive neoplasms is associated with lower morbidlity and mortality.
